# Supplementary material for: The Extract of Corydalis yanhusuo Prevents Morphine Tolerance and Dependence
Source: Pharmaceuticals (Basel). 2021 Oct 12;14(10):1034. doi: 10.3390/ph14101034 (PMC8540887; doi:10.3390/ph14101034)
Supplement: Supplementary file 1 [file pharmaceuticals-14-01034-s001.zip › pharmaceuticals-1407135-supplementary.pdf]

Materials and Methods:

The mice used in the tolerance reversal assay were also used to assess withdrawal behaviors shown in Figure S2. The withdrawal assay was carried out as previously described in the manuscript.

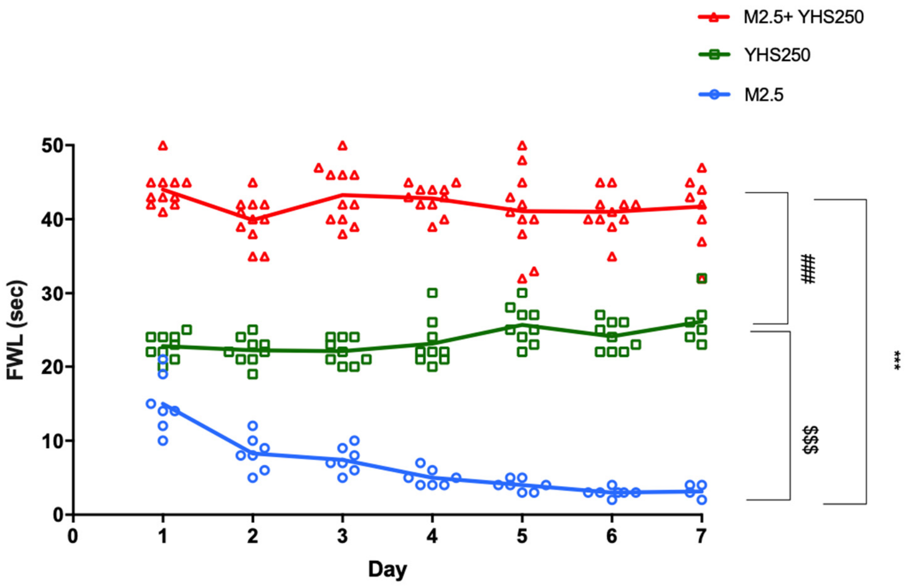

Figure S1: FWL at 30 min after morphine 2.5 mg/kg (M2.5), YHS (250 mg/kg), and morphine +YHS (M2.5 + YHS) over a period of 7 days (n = 7–11, i.p. administration) to display tolerance. Two way ANOVA revealed significant drug tolerance of morphine (M2.5) over 7 days (D) $F(6,168) = 7.023$ $p < 0.0001$, followed by Tukey's multiple comparison test. Two way ANOVA revealed significant analgesic effects between M2.5, YHS 250, and M2.5+YHS $F(6,168) = 7.023$ $p < 0.0001$, followed by Tukey's multiple comparison test M2.5*** $p < 0.0001$ compared to YHS on D1–7, YHS ### $p < 0.0001$ compared to M2.5 + YHS on D1–7, M2.5 $$$ $p < 0.001$ compared to M2.5 + YHS on D1–7.

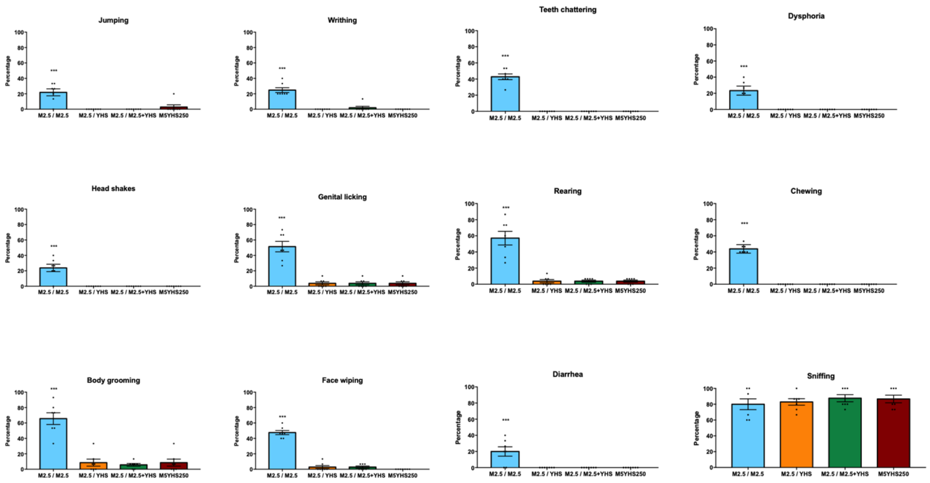

Figure S2: Jumping, writhing, head shakes, genital licking, body grooming, face wiping, teeth chattering, dysphoria, rearing, chewing, diarrhea, sniffing after naloxone i.p. administration (n = 7). One way ANOVA revealed significant percentage of jumping, writhing, head shakes, genital licking, body grooming, face wiping, teeth chattering, dysphoria, rearing, chewing, and diarrhea in animals treated with morphine only, F (3, 24) = 81.05 $p < 0.0001$, followed by Tukey's multiple comparison test, *** $p < 0.0001$, M2.5 *** $p < 0.0001$ compared with M2.5-YHS and M2.5-M2.5 + YHS.
